# Supplementary material for: Bioluminescence Imaging of Potassium Ion Using a Sensory Luciferin and an Engineered Luciferase
Source: J Am Chem Soc. 2024 May 3;146(19):13406–16. doi: 10.1021/jacs.4c02473 (PMC11100015; doi:10.1021/jacs.4c02473)
Supplement: Supplementary file 1 — ja4c02473_si_001.pdf [file ja4c02473_si_001.pdf]

Supporting Information

Bioluminescence Imaging of Potassium Ion Using a Sensory Luciferin and an Engineered Luciferase

Shengyu Zhao,[1,2,3‡] Ying Xiong,[1,2‡] Ranganayakulu Sunnapu,[1,2] Yiyu Zhang,[1,2] Xiaodong Tian,[1,2] and Hui-wang Ai[1,2,3,4*]

[1] Department of Molecular Physiology and Biological Physics, University of Virginia School of Medicine, Charlottesville, Virginia 22908, USA.

[2] Center for Membrane and Cell Physiology, University of Virginia School of Medicine, Charlottesville, Virginia 22908, USA.

[3] Department of Chemistry, University of Virginia, Charlottesville, Virginia 22904, USA.

[4] The UVA Comprehensive Cancer Center, University of Virginia, Charlottesville, Virginia 22908, USA.

‡These two authors contributed equally to this work.

*Corresponding author. Email: huiwang.ai@virginia.edu

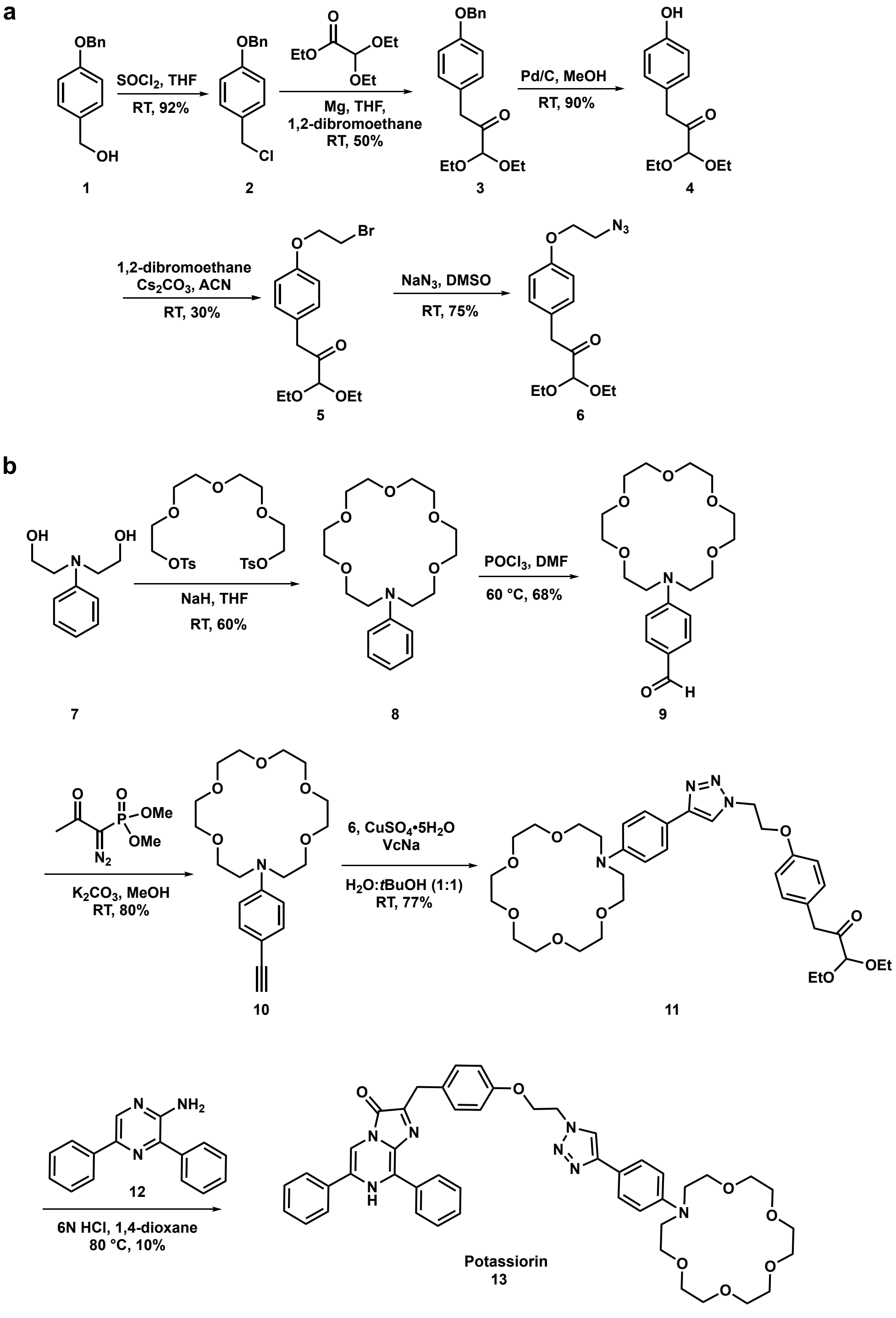

Figure S1. Synthetic route to chemically synthesize potassiorin. (**a**) Preparation of 3-(4-(2-azidoethoxy)phenyl)-1,1-diethoxypropan-2-one (**6**) from 4-benzyloxybenzyl alcohol (**1**). (**b**) Preparation of potassiorin from N-phenyldiethanolamine (**7**) and **6**.

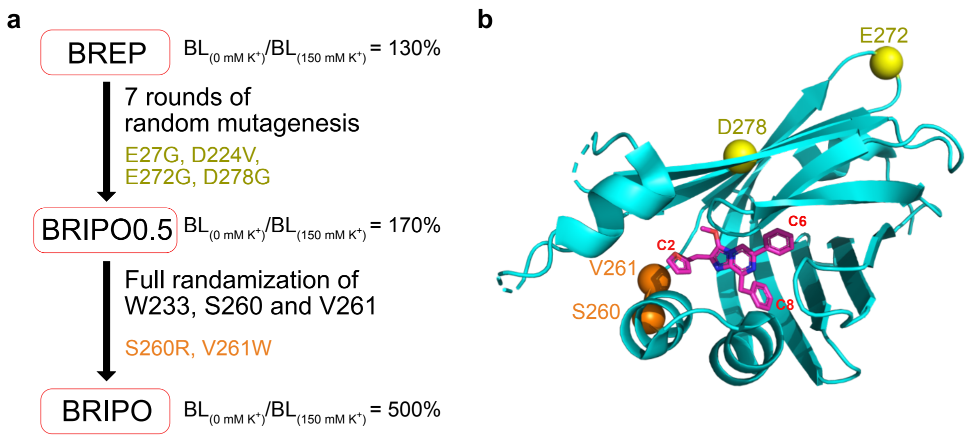

Figure S2. Engineering of BRIPO and illustration of key mutations. (**a**) Flowchart for the protein engineering process with mutations and dynamic ranges (determined with cell lysates) highlighted. (**b**) Structure of NanoLuc bound with an inactive 3-methoxy-furimazine luciferin analog (Protein Data Bank entry 7SNT). Residues in the luciferase domain that are mutated in BRIPO are also indicated. BL, bioluminescence.

	1	2	3	4	5	6	7	8	9	10	11	12	13	14	15	16	17	18	19	20	21	22	23	24	25	26	27	28	29	30
**BREP**	M	V	S	K	G	E	A	V	I	K	E	F	M	R	F	K	V	H	M	E	G	S	M	N	G	H	E	F	E	I
**BRIPO0.5**	M	V	S	K	G	E	A	V	I	K	E	F	M	R	F	K	V	H	M	E	G	S	M	N	G	H	G	F	E	I
**BRIPO**	M	V	S	K	G	E	A	V	I	K	E	F	M	R	F	K	V	H	M	E	G	S	M	N	G	H	G	F	E	I

	31	32	33	34	35	36	37	38	39	40	41	42	43	44	45	46	47	48	49	50	51	52	53	54	55	56	57	58	59	60
**BREP**	E	G	E	G	E	G	R	P	Y	E	G	T	Q	T	A	K	L	K	V	T	K	G	G	P	L	P	F	S	W	D
**BRIPO0.5**	E	G	E	G	E	G	R	P	Y	E	G	T	Q	T	A	K	L	K	V	T	K	G	G	P	L	P	F	S	W	D
**BRIPO**	E	G	E	G	E	G	R	P	Y	E	G	T	Q	T	A	K	L	K	V	T	K	G	G	P	L	P	F	S	W	D

	61	62	63	64	65	66	67	68	69	70	71	72	73	74	75	76	77	78	79	80	81	82	83	84	85	86	87	88	89	90
**BREP**	I	L	S	P	Q	F	M	Y	G	S	R	A	F	I	K	H	P	A	D	I	P	D	Y	Y	K	Q	S	F	P	E
**BRIPO0.5**	I	L	S	P	Q	F	M	Y	G	S	R	A	F	I	K	H	P	A	D	I	P	D	Y	Y	K	Q	S	F	P	E
**BRIPO**	I	L	S	P	Q	F	M	Y	G	S	R	A	F	I	K	H	P	A	D	I	P	D	Y	Y	K	Q	S	F	P	E

	91	92	93	94	95	96	97	98	99	100	101	102	103	104	105	106	107	108	109	110	111	112	113	114	115	116	117	118	119	120
**BREP**	G	F	K	W	E	R	V	M	N	F	E	D	G	G	A	V	T	V	T	Q	D	T	S	L	E	D	G	T	L	I
**BRIPO0.5**	G	F	K	W	E	R	V	M	N	F	E	D	G	G	A	V	T	V	T	Q	D	T	S	L	E	D	G	T	L	I
**BRIPO**	G	F	K	W	E	R	V	M	N	F	E	D	G	G	A	V	T	V	T	Q	D	T	S	L	E	D	G	T	L	I

	121	122	123	124	125	126	127	128	129	130	131	132	133	134	135	136	137	138	139	140	141	142	143	144	145	146	147	148	149	150
**BREP**	Y	K	V	K	L	R	G	T	N	F	P	P	D	G	P	V	M	Q	K	K	T	M	G	W	E	A	S	T	E	R
**BRIPO0.5**	Y	K	V	K	L	R	G	T	N	F	P	P	D	G	P	V	M	Q	K	K	T	M	G	W	E	A	S	T	E	R
**BRIPO**	Y	K	V	K	L	R	G	T	N	F	P	P	D	G	P	V	M	Q	K	K	T	M	G	W	E	A	S	T	E	R

	151	152	153	154	155	156	157	158	159	160	161	162	163	164	165	166	167	168	169	170	171	172	173	174	175	176	177	178	179	180
**BREP**	L	Y	P	E	D	G	V	L	K	G	D	I	K	M	A	L	R	L	K	D	G	G	R	Y	L	A	D	F	K	T
**BRIPO0.5**	L	Y	P	E	D	G	V	L	K	G	D	I	K	M	A	L	R	L	K	D	G	G	R	Y	L	A	D	F	K	T
**BRIPO**	L	Y	P	E	D	G	V	L	K	G	D	I	K	M	A	L	R	L	K	D	G	G	R	Y	L	A	D	F	K	T

	181	182	183	184	185	186	187	188	189	190	191	192	193	194	195	196	197	198	199	200	201	202	203	204	205	206	207	208	209	210
**BREP**	T	Y	K	A	K	K	P	V	Q	M	P	G	A	Y	N	V	D	R	K	L	D	I	T	S	H	N	E	D	Y	T
**BRIPO0.5**	T	Y	K	A	K	K	P	V	Q	M	P	G	A	Y	N	V	D	R	K	L	D	I	T	S	H	N	E	D	Y	T
**BRIPO**	T	Y	K	A	K	K	P	V	Q	M	P	G	A	Y	N	V	D	R	K	L	D	I	T	S	H	N	E	D	Y	T

	211	212	213	214	215	216	217	218	219	220	221	222	223	224	225	226	227	228	229	230	231	232	233	234	235	236	237	238	239	240
**BREP**	V	V	E	Q	Y	E	R	S	E	G	R	H	L	D	T	L	E	D	F	V	G	D	W	R	Q	T	A	G	Y	N
**BRIPO0.5**	V	V	E	Q	Y	E	R	S	E	G	R	H	L	V	T	L	E	D	F	V	G	D	W	R	Q	T	A	G	Y	N
**BRIPO**	V	V	E	Q	Y	E	R	S	E	G	R	H	L	V	T	L	E	D	F	V	G	D	W	R	Q	T	A	G	Y	N

	241	242	243	244	245	246	247	248	249	250	251	252	253	254	255	256	257	258	259	260	261	262	263	264	265	266	267	268	269	270
**BREP**	L	S	Q	V	L	E	Q	G	G	V	S	S	L	F	Q	N	L	G	V	S	V	T	P	I	Q	R	I	V	L	S
**BRIPO0.5**	L	S	Q	V	L	E	Q	G	G	V	S	S	L	F	Q	N	L	G	V	S	V	T	P	I	Q	R	I	V	L	S
**BRIPO**	L	S	Q	V	L	E	Q	G	G	V	S	S	L	F	Q	N	L	G	V	R	W	T	P	I	Q	R	I	V	L	S

	271	272	273	274	275	276	277	278	279	280	281	282	283	284	285	286	287	288	289	290	291	292	293	294	295	296	297	298	299	300
**BREP**	G	E	N	G	L	K	I	D	I	H	V	I	I	P	Y	E	G	L	S	G	D	Q	M	G	Q	I	E	K	I	F
**BRIPO0.5**	G	G	N	G	L	K	I	G	I	H	V	I	I	P	Y	E	G	L	S	G	D	Q	M	G	Q	I	E	K	I	F
**BRIPO**	G	G	N	G	L	K	I	G	I	H	V	I	I	P	Y	E	G	L	S	G	D	Q	M	G	Q	I	E	K	I	F

	301	302	303	304	305	306	307	308	309	310	311	312	313	314	315	316	317	318	319	320	321	322	323	324	325	326	327	328	329	330
**BREP**	K	V	V	Y	P	V	D	N	H	H	F	K	V	I	L	H	Y	G	T	L	V	I	D	G	V	T	P	N	M	I
**BRIPO0.5**	K	V	V	Y	P	V	D	N	H	H	F	K	V	I	L	H	Y	G	T	L	V	I	D	G	V	T	P	N	M	I
**BRIPO**	K	V	V	Y	P	V	D	N	H	H	F	K	V	I	L	H	Y	G	T	L	V	I	D	G	V	T	P	N	M	I

	331	332	333	334	335	336	337	338	339	340	341	342	343	344	345	346	347	348	349	350	351	352	353	354	355	356	357	358	359	360
**BREP**	D	Y	F	G	R	P	Y	E	G	I	A	V	F	D	G	K	K	I	T	V	T	G	T	L	W	N	G	N	K	I
**BRIPO0.5**	D	Y	F	G	R	P	Y	E	G	I	A	V	F	D	G	K	K	I	T	V	T	G	T	L	W	N	G	N	K	I
**BRIPO**	D	Y	F	G	R	P	Y	E	G	I	A	V	F	D	G	K	K	I	T	V	T	G	T	L	W	N	G	N	K	I

	361	362	363	364	365	366	367	368	369	370	371	372	373	374	375	376	377	378	379	380	381	382	383	384	385	386	387	388	389	390
**BREP**	I	D	E	R	L	I	N	P	D	G	S	L	L	F	R	V	T	I	N	G	V	T	G	W	R	L	H	E	R	I
**BRIPO0.5**	I	D	E	R	L	I	N	P	D	G	S	L	L	F	R	V	T	I	N	G	V	T	G	W	R	L	H	E	R	I
**BRIPO**	I	D	E	R	L	I	N	P	D	G	S	L	L	F	R	V	T	I	N	G	V	T	G	W	R	L	H	E	R	I

	391	392
**BREP**	L	A
**BRIPO0.5**	L	A
**BRIPO**	L	A

Figure S3. Sequence alignment of BREP, BRIPO0.5 and BRIPO. Sequences derived from mScarlet-I and teLuc are colored in red and cyan, respectively. Linker residues are colored in gray. Mutations gained during protein engineering are shaded in yellow (from random mutagenesis) and orange (from site-directed randomization).

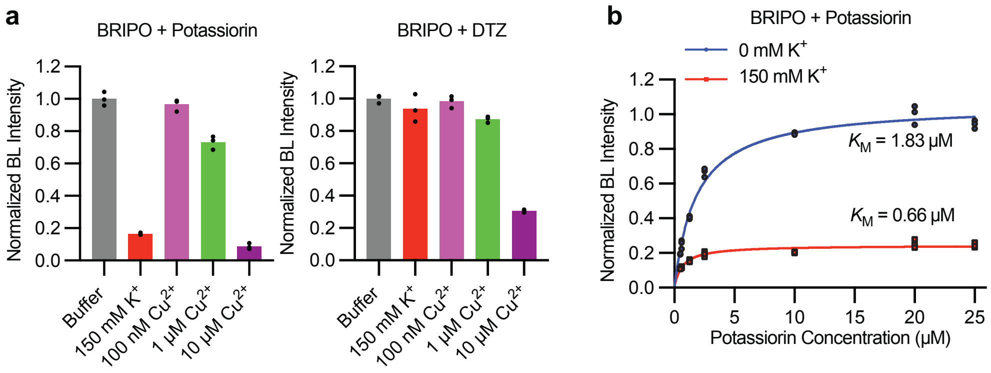

Figure S4. Additional *in vitro* characterization of BRIPO. (**a**) Bioluminescence responses of BRIPO in the presence of potassiorin (left) or DTZ (right) to 150 mM K^+ and the indicated concentrations of Cu^{2+}. n = 3 technical repeats. (**b**) Potassiorin concentration dependency of BRIPO bioluminescence at 590 nm in the presence or absence of 150 mM KCl. n = 3 technical repeats. The Michaelis-Menten equation was used to fit the data and derive the Michaelis constants (K_M). BL, bioluminescence.

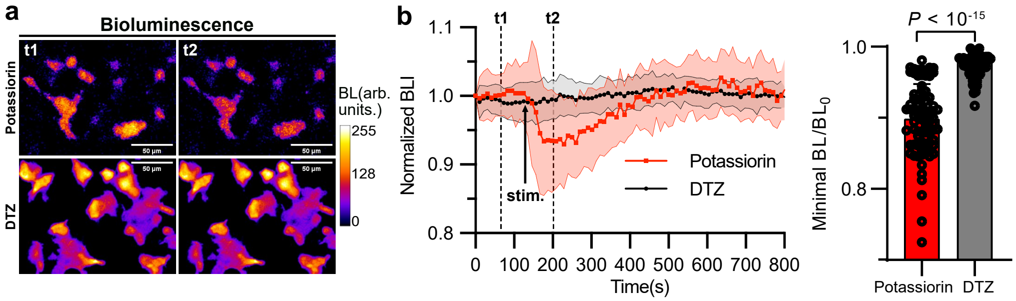

Figure S5. Imaging K^+ influx into HEK 293T cells in high K^+ buffer. (**a**) Representative pseudocolored bioluminescence images of BRIPO-expressing HEK 293T cells in a high K^+ (200 mM) buffer in the presence of potassiorin (top) or DTZ (bottom) before (left) and after (right) treatment with a combination of nigericin, ouabain, and bumetanide. Scale bar: 50 μm. (**b**) Quantification of bioluminescence intensity changes of individual cells from experiments in panel a. Data are presented as mean ± s.d. (n = 85 cells for the potassiorin group, n = 85 cells for the DTZ group).

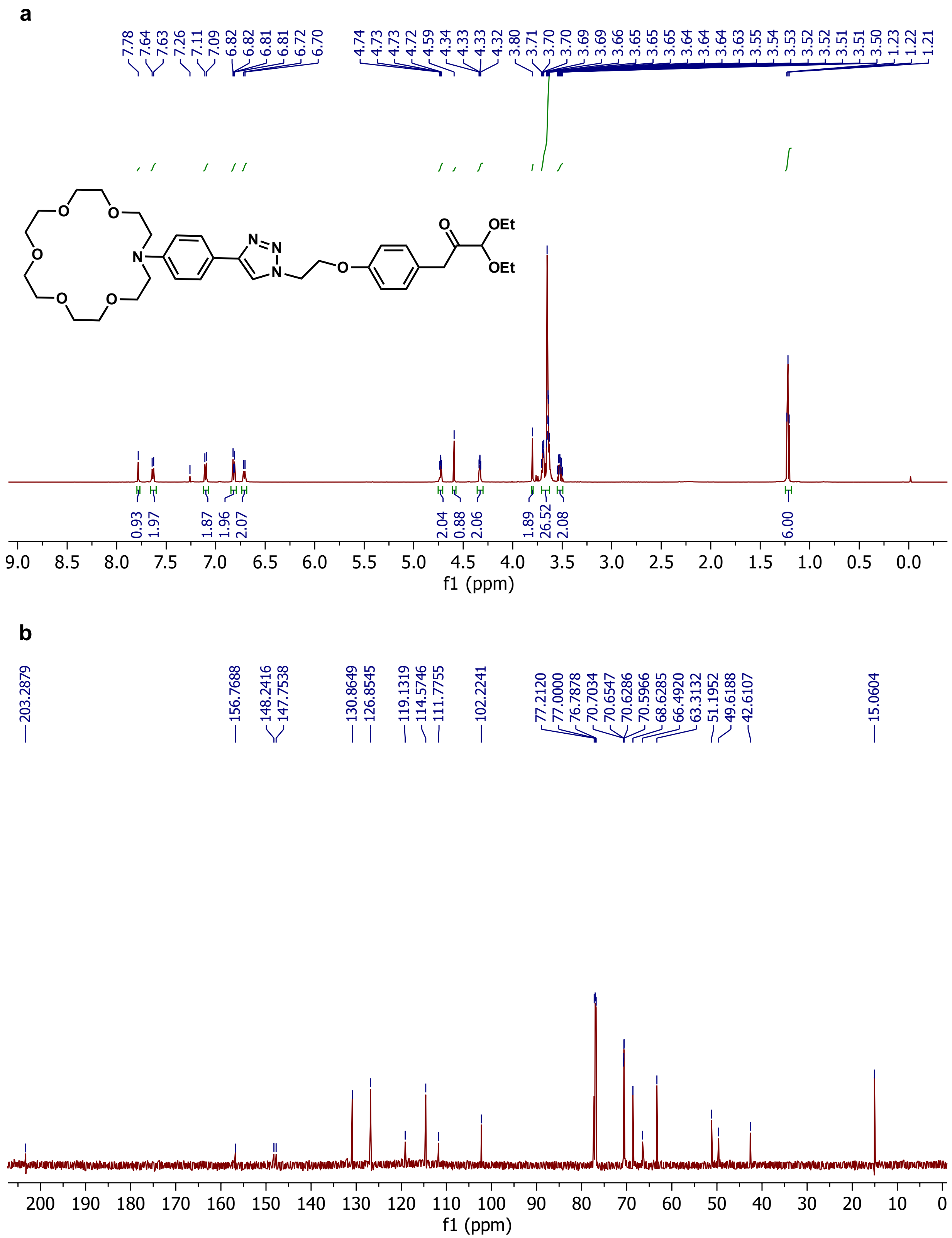

Figure S6. ^{1}**H-NMR (a) and** ^{13}**C-NMR (b) spectra for compound 11.**

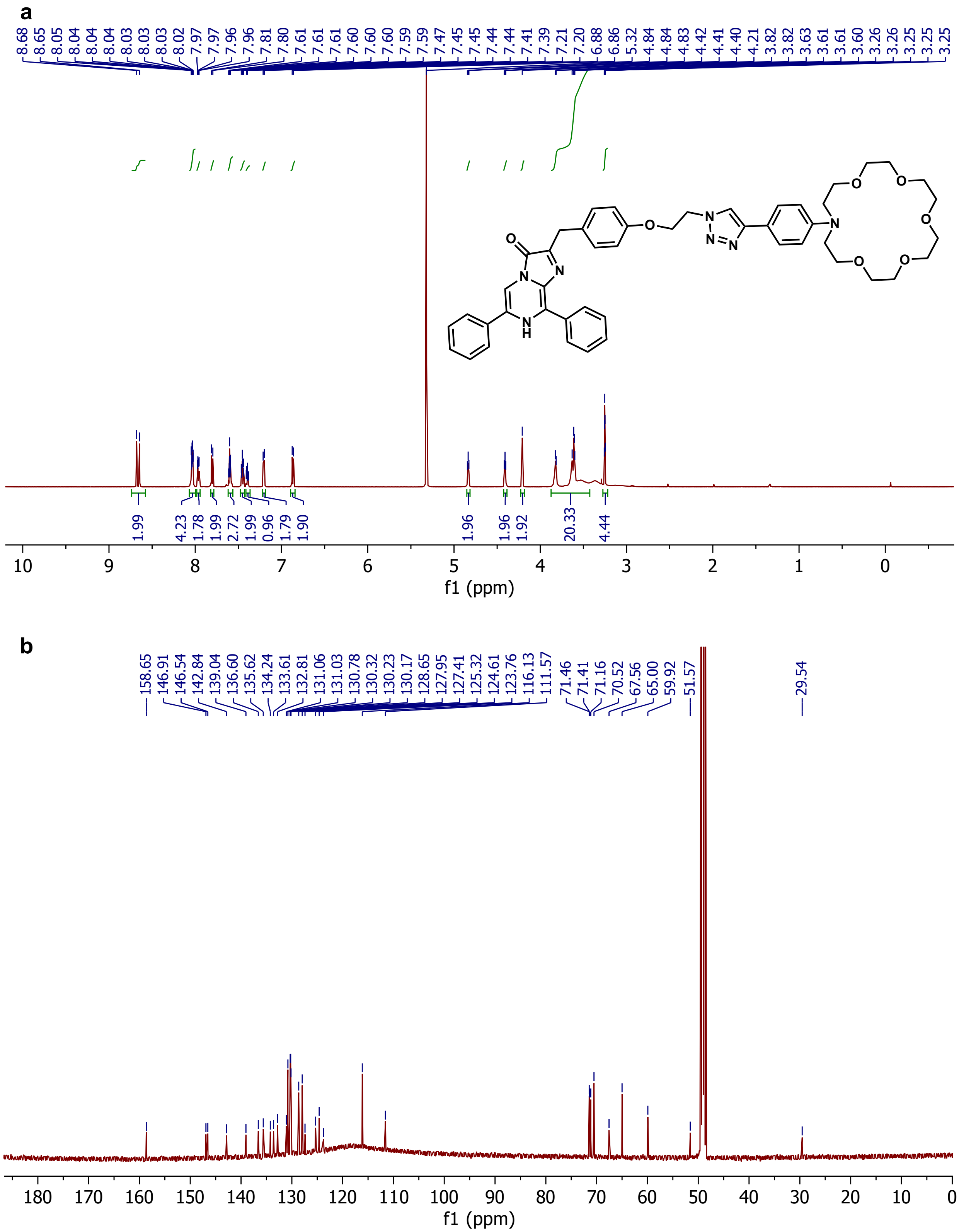

Figure S7. **^{1}H-NMR (a) and ^{13}C-NMR (b) spectra for potassiorin (compound 13).**

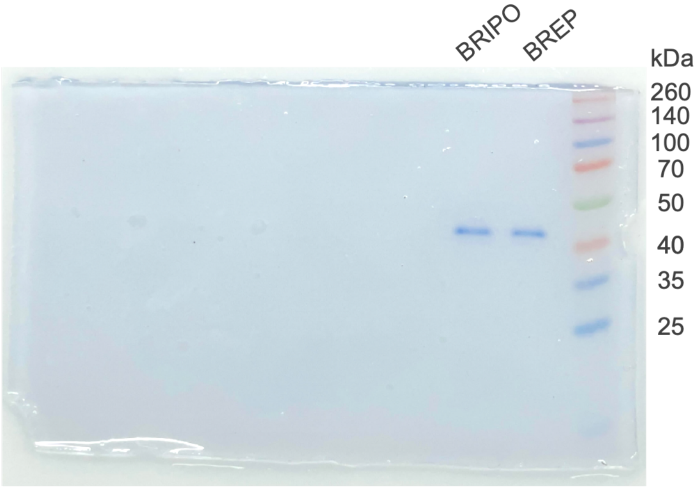

Figure S8. SDS-PAGE showing the purity of the prepared BREP and BRIPO proteins.

Table S1. Apparent Michaelis constants (K_M), relative maximal photon production rates (V_{max}), and quantum yield (QY) of the BRIPO-potassiorin pair in the presence or absence of 150 mM K^+.

BRIPO-potassiorin	K_M (μM)	Relative V_{max}	QY (%)
+ KCl	0.66	1	1.75
− KCl	1.83	4.33	2.01

Movie S1.
Description: BLI of BRIPO-expressing HEK 293T cells in low K^+ buffer in response to a combination of nigericin, ouabain, and bumetanide.

Movie S2.
Description: BLI of BRIPO-expressing HEK 293T cells stably expressing mTrek and several other ion channels in response to arachidonic acid.

Movie S3.
Description: BLI of a mouse with BRIPO expression in the brain in response to local glutamate stimulation.
